# Characterization of Visual Symptomatology Associated with Refractive, Accommodative, and Binocular Anomalies

**DOI:** 10.1155/2015/895803

**Published:** 2015-08-16

**Authors:** Pilar Cacho-Martínez, Mario Cantó-Cerdán, Stela Carbonell-Bonete, Ángel García-Muñoz

**Affiliations:** Departamento de Óptica, Farmacología y Anatomía, Universidad de Alicante, 03080 Alicante, Spain

## Abstract

*Purpose*. To characterize the symptomatology of refractive, accommodative, and nonstrabismic binocular dysfunctions and to assess the association between dysfunctions and symptoms. *Methods*. 175 randomised university students were examined. Subjects were given a subjective visual examination with accommodative and binocular tests, evaluating their symptomatology. Accommodative and binocular dysfunctions (AD, BD) were diagnosed according to the number of existing clinical signs: suspect AD or BD (one fundamental clinical sign), high suspect (one fundamental + 1 complementary clinical sign), and definite (one fundamental + 2 or more complementary clinical signs). A logistic regression was conducted in order to determine whether there was an association between dysfunctions and symptoms. *Results*. 78 subjects (44.6%) reported any kind of symptoms which were grouped into 18 categories, with “visual fatigue” being the most frequent (20% of the overall complaints). Logistic regression adjusted by the presence of an uncorrected refractive error showed no association between any grade of AD and symptoms. Subjects with BD had more likelihood of having symptoms than without dysfunction group (OR = 3.35), being greater when only definite BD were considered (OR = 8.79). *Conclusions*. An uncorrected refractive error is a confusion factor when considering AD symptomatology. For BD, the more the number of clinical signs used the greater the likelihood suffering symptoms.

## 1. Introduction

In order to maintain a clear single image when reading or doing near work, both accommodative and convergence systems must be adequate. If these mechanisms fail, leading to accommodative and/or vergence dysfunctions, subjects may complain of symptoms [1]. Several authors have reported that visual discomfort symptoms are prevalent among those subjects who have prolonged near work such as using a computer or reading [2–7] and complaints may include visual fatigue, headache, blurred vision, diplopia, loss of concentration when reading or doing near work, light sensitivity, or perceptual distortions involving letter movement and fading.

Scientific literature has revealed that accommodative and/or vergence anomalies are commonly found in clinical practice [1, 8–19] and may show different symptoms between patients [20, 21]. In this sense, several studies have focused on the measurement of visual discomfort symptoms in college students [2, 6, 22–24]. Several authors have related these dysfunctions to problems with reading or academic performance [25, 26]. Others [1, 27] have reported that these visual symptoms may have a negative effect on school performance or reading comprehension, even leading to avoidance of near work. It has also been shown [28] that children with more visual symptoms have lower academic achievement than those with fewer visual symptoms. And current clinical trials about convergence insufficiency have shown the improvement of academic behaviors [29] and the decrease in performance-related symptoms [30] after successful treatment.

In any case, although in the scientific literature we can see that the authors consider the presence of symptoms essential to diagnose accommodative and/or binocular anomalies [26, 31–42], it has been shown in a scoping review [43] considering articles since the last 30 years that there is no consensus as to which symptoms should be considered in the diagnosis of each of these anomalies. In this sense, few symptoms are specific to each entity and many of them overlap when considering an accommodative or binocular dysfunction, so that this information may influence clinical management as it is difficult to associate a particular dysfunction with a particular symptom. But also many of symptoms related to accommodative and binocular disorders may not be different from those related to other conditions as visual stress (or Meares-Irlen syndrome) [5, 44] or from those related to uncorrected refractive anomalies [45].

Under these premises, the aim of this study was to characterize the symptomatology of refractive, accommodative, and nonstrabismic binocular dysfunctions in a randomised sample of university students. A further aim was to assess the association between the various dysfunctions diagnosed and the symptoms reported by the patients.

## 2. Materials and Methods

This study is part of a larger research which intended to analyse the visual health of university students by means of analyzing the prevalence of accommodative and binocular dysfunctions of the university population of University of Alicante and to characterize their symptoms. This paper is related to the characterization of symptoms.

We conducted a prospective study of a population sample of university students from the University of Alicante, selected by means of simple random sampling of all students in all years of the degree courses taught at the University of Alicante. Simple random sampling was performed of the 26,326 students attending the University of Alicante at the time when the study was conducted. The randomization was done by the Computing Service of the University. Sample size was calculated by assuming an overall prevalence of accommodative and nonstrabismic binocular dysfunctions of 25% [11, 12, 18] with a confidence level of 95%, requiring a 5% for the proportion's estimation and a dropout rate of 20%, yielding a sample of 357 undergraduate and postgraduate students. As a requirement for obtaining data, students had to be aged between 18 and 35 years old. Establishing the upper age limit of 35 years was to avoid including prepresbyopic subjects in the study [46] who could bias the diagnosis of an accommodative and nonstrabismic binocular disorder.

Participants were initially contacted via an email informing them that they had been randomly selected from the university student population to participate in this research. Due to the low response rate yielded by this approach, it was decided to contact them by telephone to inform them of the study. Despite multiple attempts to contact students via email and telephone, there were 177 students who finally participated in the study, representing a response rate of 49.6%. There were a variety of reasons for nonresponse: 131 students did not respond either to emails or telephone calls, 6 lived elsewhere, 21 did not want to participate, and 20 students agreed to participate but did not attend their appointment.

This study was conducted in accordance with the Declaration of Helsinki, and the Ethics Committee of University of Alicante approved the study. All subjects gave their written informed consent once the nature of the study had been explained. All of the 177 students were given a visual examination, which besides a visual health examination and a refractive examination also included specific tests to determine the subject's accommodative and binocular status.

Visual examination consisted of the following procedures.

- *Sociodemographic Data.* Besides personal details, we also collected academic data such as number of hours of study per day and academic performance, this latter being calculated as the number of credits obtained divided by the number of credits studied in the last academic year.
- *Clinical History.* Data were collected on the symptoms reported by the subjects. To this end, we did not use a questionnaire but we used the categories of symptoms described by García-Muñoz et al. [43] in a recent scoping review, in which they indicated the different types of symptoms which may be associated with accommodative and binocular dysfunction. We showed the list of 34 symptoms categories and patients were asked if they were suffering any type of these symptoms related to their vision. Several symptoms were fully explained to ensure understanding, for example, the symptom of “visual fatigue” (which refers specifically to symptoms described by patients such as tired eyes, eyestrain, or eye fatigue). Subjects were considered to present a visual symptomatology when they reported one or more symptoms. For the purpose of this analysis, a symptom was considered present when it was either the patients' main concern or if it occurred frequently, so that feeling the symptom once was not enough to render a positive answer.
- *Refractive Examination.* This was performed by means of static retinoscopy and a subjective examination. The subjective examination was performed by means of monocular fogging method with cross-cylinder followed by binocular balancing to a standard endpoint of maximum plus for best visual acuity. Once the maximum plus value for best visual acuity had been obtained, this result of the subjective examination was used as the baseline for all accommodative and binocular tests.
- *Accommodative and Binocular Tests.* The following tests were performed. Phoria measurement was done with cover test. Unilateral and alternate cover test measurements [47–49] were done for distant and near vision while the subject was instructed to fixate on a single letter of 20/30 visual acuity. Using a prism bar, the deviation value was midway between the low and high neutral findings using an alternate cover test. Measurement of gradient AC/A ratio [50] was done using the cover test and −1.00 D lenses. We calculated AC/A ratio [50]. Monocular estimate method (MEM) dynamic retinoscopy [51] at 40 cm was done with the result of the subjective exam placed in a trial frame and using trial lenses. Monocular accommodative amplitude was measured by Donder's push-up method [52] using a single 20/30 Snellen line target in free space. The target was slowly moved towards the patient until reported blurring, and the distance from this point to the spectacle plane was then recorded in diopters. Monocular and binocular accommodative facility were measured following the procedure of Zellers et al. [53] at 40 cm and using ±2.00 D flipper lenses and the 20/30 letter line on Vectogram 9 (Bernell) which includes a suppression control for the binocular measurement. Monocular accommodative facility was measured in the same manner but without polarized glasses and with the nonviewing eye occluded. Both monocular and binocular accommodative facility were measured during one minute and the cycles per minute (cpm) were recorded, evaluating if the patient had difficulty in focusing with plus or minus lenses. Near point of convergence was measured in free space using an accommodative target of 20/30 visual acuity at 40 cm [54] and moving the target away from the subject at a speed of approximately 1 to 2 cm per second [55] until the break and recovery findings. Distance was calculated from the midsagittal plane of the patients' head to the nearest half centimeter. Positive and negative relative accommodations were measured with plus and minus, respectively, behind the phoropter, and using an accommodative target of 20/30 visual acuity at 40 cm [50] until sustained blur was detected. Positive and negative fusional vergences were determined at far and near vision with phoropter's Risley prism (with a smooth gradual increase in prism power) using an accommodative target of 20/30 visual acuity and performed according to the subject's type of heterophoria (for exophoria the positive fusional vergence was measured first and the negative fusional vergence was first performed for esophoria) [56, 57]. Vergence facility was measured in free space using an accommodative target of 20/30 visual acuity at 40 cm, with the prism combination of 3 Δ base-in/12 Δ base-out [58, 59] and evaluating if the patient had difficulty in fusing with the base-in or base-out prism. Determination of fusion was done by means of the 4-dot Worth test [50] and stereopsis was measured using graded circles of Randot SO-002 test [60] in free space.

Once all these tests had been performed, the results were analysed in order to determine the existence of refractive, accommodative, and/or binocular dysfunctions. In order to avoid bias, diagnosis of each dysfunction was completed by two authors different to the person who performed the visual examination, so that the persons who determined the diagnoses were masked to the symptoms.

The inclusion criteria for the study were applied to these initial data and consisted of having a visual acuity of 20/20 with the best correction, the absence strabismus or amblyopia, and any ocular or systemic disease that might affect the results. Two students presented an amblyopia and strabismus so that the final number of subjects included in the study was 175, of whom 59 were male (33.7%) and 116 female (66.3%), with a mean age of 22.90 ± 3.96 years.

Patients were diagnosed with refractive dysfunction when a difference was detected between their habitual refraction and their subjective examination results obtained in this study. The clinical criteria applied to establish this difference were the following:

- A less negative subjective examination result than the habitual refraction; in other words, the patient was overcorrected for myopia (equal to or greater than 0.50 D).
- Changes in the sphere or cylinder equal to or greater than 0.50 D, with which visual acuity was increased by at least one line with the new refraction.

Thus, subjects were classified into two refractive categories as follows:

- In subjects with an uncorrected refractive error, the patient used a prescription different to that indicated by the subjective examination, or the patient did not use a prescription but needed it.
- In subjects without an uncorrected refractive error, the patient's habitual refraction was satisfactory.

Accommodative dysfunctions (AD) and binocular dysfunctions (BD) were diagnosed in accordance with the criteria described in the literature [42, 50]. However, since there is no enough scientific evidence to support the use of any given diagnostic criterion to accurately define each dysfunction [21], we decided to classify dysfunctions on the basis of the number of clinical signs associated with each dysfunction, classifying the signs that could be associated with each dysfunction as fundamental and complementary. The classification employed was as follows:

- Suspect AD or BD: one fundamental clinical sign.
- High suspect AD or BD: one fundamental clinical sign + 1 complementary clinical sign.
- Definite AD or BD: one fundamental clinical sign + 2 or more complementary clinical signs.

Table 1 gives the fundamental and complementary clinical signs used in this study to diagnose each accommodative and binocular dysfunction. For binocular conditions we used the classification made by Scheiman and Wick in which the calculated AC/A ratio is considered to diagnose a particular binocular condition. Following this classification high AC/A ratio conditions are convergence excess and divergence excess, low AC/A ratio includes convergence insufficiency and divergence insufficiency, and normal AC/A ratio conditions refer to fusional vergence dysfunction (also known as binocular instability [61]), basic esophoria, and basic exophoria. With these considerations, patients were grouped into different groups: patients with accommodative dysfunctions (AD), binocular dysfunctions (BD), and both accommodative and binocular anomalies (AD + BD).

Subjects who only presented an uncorrected refractive error were considered as the group of refractive dysfunction (RD).

And those subjects, who did not present any refractive, accommodative, or binocular dysfunction, were considered as the group named without dysfunction (WD).

### 2.1. Data Analysis

An analysis of the relationships between sociodemographic variables and the presence of symptoms was conducted using the samples as independent variables, using the chi-square test for categorical variables, the Mann-Whitney *U* test for continuous variables, and the Rho Spearman for correlation analysis. Significance level was 0.05 for all analysis.

Once the subjects had been classified according to the refractive, accommodative, or binocular dysfunction they presented, we analyzed their symptoms and the dysfunction diagnosed, conducting a logistic regression in order to determine whether there was an association between each dysfunction and the symptoms reported by the participants. This procedure yielded the raw odds ratio (OR). The presence of an uncorrected refractive error was considered a confounding variable, as it was associated with both the existence of symptoms and the presence of AD and BD (*p* < 0.05). We thus determined the probability of having visual symptoms according to the dysfunctions diagnosed after having eliminated the effect that uncorrected refractive errors could have on this relationship and obtained an adjusted estimate of the association between the two variables by means of the adjusted odds ratio (OR).

Statistical analysis was performed using SPSS 20.0 for Windows.

## 3. Results

Of the 175 study participants, 61 subjects had some form of accommodative and/or binocular dysfunctions, 59 patients were classified as refractive dysfunction, and 55 subjects were grouped in the group without dysfunction. Furthermore, of the 61 subjects with some form of accommodative and/or binocular anomalies, 33 of them also presented an uncorrected refractive error but they were classified as belonging to the accommodative or binocular dysfunction group.

Of the 175 subjects examined, 78 people (44.6%) reported having some kind of visual symptoms which were grouped into 18 different categories.  Table 2 shows these categories encountered, together with the number of complaints reported by the patients.

An analysis of the sociodemographic data for the 175 university students revealed that there was no association between having symptoms (in general) and wearing contact lenses (*p* = 0.44), sex (*p* = 0.17), number of hours of study per day (*p* = 0.068), or students' academic performance (*p* = 0.21). When this analysis was done considering each type of symptoms isolated, there was only an association between the symptom of blurred vision and the number of hours per day (*p* = 0.003), so that patients with blurred vision studied more number of hours per day compared with patients without symptoms. Correlation analysis showed no correlation between the number of symptoms and the number of hours of study per day (*r* = 0.13, *p* = 0.084) or academic performance (*r* = 0.13, *p* = 0.095).

Tables 3, 4, 5, and 6 show the categories of symptoms detected in the different group of patients, without dysfunction, refractive, accommodative, and/or binocular dysfunctions.

Lastly, Table 7 shows the results of the logistic regression performed to determine the association between visual symptoms and the different dysfunctions diagnosed and gives the raw and adjusted OR for the presence of an uncorrected refractive error. This analysis was conducted for AD and BD dysfunctions both in general and according to the number of clinical signs present (suspect, high suspect, and definite). Only relationships with a statistically significant raw or adjusted OR (*p* < 0.05) are shown.

## 4. Discussion

The results of this study of a university population selected by means of random sampling indicate the presence of different symptoms in subjects diagnosed with refractive, accommodative, and binocular dysfunctions. We found an association between visual symptomatology and binocular dysfunction, whereby the higher the number of clinical signs used to diagnose binocular dysfunction, the greater the likelihood of symptoms.

These findings, however, present some limitations. Despite being a randomised sample, our sample size was not large and this may have led to bias in the results. The AD and BD groups were sometimes small, which could lead to a statistical type II error; that is, no statistically significant differences appear when in fact they might exist in a larger sample. In addition to that, the small sample size makes it difficult to establish that this sample is representative of the whole university population. For that reason, results of this study only may be applied to the population analyzed and cannot be extrapolated to the general university population.

Another important consideration when analyzing the results of this research is the diagnostic criteria we used which are different to other studies. Several authors have used different approaches for diagnosing an anomaly, from requiring patients to fail a test on two occasions [62] to use an algorithm to evaluate the degree of decompensated heterophoria [63, 64]. Scientific evidence has shown that there are different diagnostic criteria for these dysfunctions, showing that for several disorders some tests are more important than others [21]. This systematic review considering articles since the last 26 years [21] has shown for binocular conditions that the authors use different clinical criteria based on their own criteria, but there is a lack of studies which have evaluated the diagnostic accuracy (with data of predictive values, sensitivity and specificity, and ROC analysis) of clinical signs used for these anomalies. So the authors usually reach their diagnoses on the basis of the criteria they consider but without any explanation about why several clinical signs are used and others are not. In this sense, all authors consider for binocular disorders the measurement of the deviation, although other tests as NPC for convergence insufficiency are not used by all of them. The same happens with other binocular dysfunctions with large differences between authors although all of them agree with the measurement of the deviation. To date, there is only one study [42] which has evaluated the diagnostic validity for clinical signs associated with a high near exophoria, but it is necessary to explore more these data with greater samples and in other populations. For this reason, we considered the phoria measurement as the fundamental clinical sign for all conditions.

For accommodative anomalies, diagnostic accuracy studies have shown that there is only certain evidence for several conditions. For accommodative insufficiency ROC analysis [26] has shown that the low accommodative amplitude is the test which has the potential discrimination ability for its diagnosis so we decided to use this clinical sign as the fundamental one. For accommodative excess the only study based on epidemiological criteria showed that a high positive relative accommodation (PRA) may be related to this anomaly [38]. However, this study had an important bias as the authors obtained the sensitivity values through the same tests previously used to diagnose the anomalies and without a ROC analysis. For this reason we decided not to use the PRA value as fundamental but only complementary. We used the fundamental clinical sign of failing monocular accommodative facility with plus lenses as this is the only monocular test which may be considered to be related to the accommodative system. The same was considered for accommodative infacility, being the test of failing monocular accommodative facility with plus and minus lenses, the fundamental clinical sign for its diagnosis.

As we can see, in the present study, we used this knowledge of scientific evidence to define which signs should be considered fundamental and which should be considered complementary. So, we created a specific methodology for classifying accommodative and binocular disorder. Some authors have performed similar classifications, diagnosing a disorder according to the number of clinical signs that the subject presented, mainly for convergence insufficiency [8, 36, 39, 40], but this is not the case for the other dysfunctions.

Results of this study reveal that there was no association between having symptoms and student's academic performance. Furthermore, students with more visual symptoms did not have lower academic achievement. These findings are different than those encountered by other authors who have shown a negative effect on school performance. Vaughn et al. [28] encountered that the higher the score in symptoms the poorer the academic performance in children. Shin et al. [1] found that children with both accommodative dysfunctions and a combination of accommodative and vergence dysfunctions had significantly lower academic scores. However they found that those patients with vergence dysfunctions alone had no relationship with academic achievement. In addition to that, several clinical trials about the treatment of convergence insufficiency in children have shown the improvement of academic behaviours [29] and a significant decrease in performance-related symptoms (reading performance and attention) [30] after treatment. Comparisons are difficult to argue due to the different population examined (children versus adults) but in any case the only association we encountered was for blurred vision and the number of hours per day, showing that patients with blurred vision studied more number of hours per day compared with patients without symptoms. However it seems that in our university subjects the symptomatology does not affect their academic performance. In this sense, for university students, Grisham et al. [27] found an association between symptoms and reading performance. Comparisons are again difficult to discuss as they analyzed the reading performance considering a patient reading after one hour and two hours of near work, and in our study we have considered the academic performance as the number of credits obtained divided by the number of credits studied in the last academic year.

Interestingly, a closer analysis of the results reveals that numerous symptoms were associated with the different dysfunctions identified. We found 18 different categories of symptoms reported by the subjects, the most prevalent of which was “visual fatigue,” mentioned in 20% of the reports.

It should also be noted that a quarter of people in the without dysfunction group (14 out of 55) presented symptoms similar to those with visual dysfunctions, such as visual fatigue, headache, or ocular pain. However, these subjects did not present any ocular or visual clinical sign which would explain this symptomatology. This finding suggests that some symptoms may be reported by patients and have no relationship with vision. In fact other authors have shown that several of these symptoms are related to other conditions as dry eye [3] and not only with a visual disorder. However as we did not test for these conditions as dry eye syndrome, we cannot explain any relationship.

As regards dysfunctions, we found that in all groups some patients presented clinical signs but did not report symptoms of any kind. This result is similar to that obtained in the study of Horwood et al. [65] which reports similar findings in a group of university students. In that study the authors used the CISS V-15 questionnaire. Although this survey was developed to monitor symptoms in persons with CI, Horwood et al. [65] used it as a screening tool to confirm the absence of significant visual symptoms. They encountered a strong mismatch between signs and symptoms. Particularly they demonstrated that symptoms often associated with convergence insufficiency (CI) are also common in young adults without clinical signs of CI and also that the majority of subjects with the clinical signs of CI had no symptoms.

Of particular interest is the fact that only the 46% (27 out of 59) of subjects with refractive dysfunction presented symptoms, even though most members of this group experienced an increase in visual acuity with the new correction. This finding suggests a deficit in the health education of these subjects, a possibility that should be taken into account by eye care professionals when conducting eye examinations. It is surprising that university students, who require greater visual demand than others, are not conscious of this visual health problem. However, it was the refractive dysfunction group which presented the highest number of symptoms, with 14 categories. Those most frequently reported were headache, visual fatigue, blurred vision, and dry or gritty eyes, symptoms typically associated with not wearing the proper correction. On the other hand, there were no complaints in the accommodative dysfunction group of difficulty in focusing from one distance to another or of headache, although in the literature such symptoms have been associated with these dysfunctions [43]. Furthermore, we did not find specific symptoms in the accommodative dysfunction or binocular dysfunction groups that were exclusively related to one dysfunction alone but rather observed that there were several categories common to both dysfunction groups, such as visual fatigue, blurred vision, or sore eyes. Therefore, one can conclude that the symptoms of visual problems are very similar in all refractive, accommodative, or binocular dysfunctions. This finding coincides with the results of the scoping review by García-Muñoz et al. [43], in which the authors found that most of the symptoms described in the scientific literature were related to both accommodative and binocular dysfunctions, although in that study the symptomatology of refractive dysfunctions was not determined.

Unadjusted associations between dysfunctions and symptoms were statistically significant in all dysfunction groups (refractive, accommodative, and binocular). The raw odds ratio indicated that those subjects with some kind of visual dysfunction were more likely to experience symptoms than those in the without dysfunction group.

However, when these associations were adjusted for the presence of an uncorrected refractive error, most of them ceased to be statistically significant, except for the group of binocular dysfunction. Particularly, for binocular dysfunctions, the odds ratio increased when high suspect and definite binocular dysfunction were considered. This implies that the more the clinical signs a subject presented, the greater the likelihood of experiencing symptoms. However this association does not mean that the more the clinical signs present the greater the frequency of symptoms as we did not examine the frequency of symptoms. In fact the study of Bade et al. [66] has shown that for children with convergence insufficiency there is no association between the severity of the clinical signs and their level of symptoms. For that reason future studies should show how this association encountered in our study may be modified if the frequency of symptoms is considered.

Our results indicate that an uncorrected refractive error contaminates the symptoms of visual dysfunction, fundamentally when an accommodative dysfunction is present. This should make us consider that when an accommodative anomaly is present and the patient has also an uncorrected refractive error, we cannot assure that patients' symptoms are due to the accommodative problem.

This influence of uncorrected refractive error suggests that when an accommodative or vergence dysfunction is detected, the refractive error should be corrected first before initiating a specific treatment to alleviate the patient's symptoms. This idea is consistent with the proposal made by Dwyer and Wick [67] who have suggested that correction of an uncorrected refractive error improves accommodative and binocular function. Nevertheless, it is unusual for studies of accommodative and binocular dysfunctions in adults to include details on how uncorrected refractive error has been managed and this may have led to research bias. Studies on prevalence of these conditions in adults [20] do not usually offer the prevalence rates about refractive dysfunctions. And even several authors exclude those subjects with refractive anomalies [11]. When considering diagnosis purposes [21] few studies refer to refractive anomalies [33, 34]. And for studies about treatment [68] only the clinical trials about convergence insufficiency [69–71] do detail the adequate prescription of refractive correction before the treatment. In future studies, researchers should consider the effect of an uncorrected refractive error on the prevalence, diagnosis, and treatment of accommodative and vergence dysfunctions to determine its influence in the management of these anomalies.

## Figures and Tables

**Table 1:** Clinical signs used in the study for diagnosing accommodative and binocular anomalies (AA: accommodative amplitude; MAF/BAF: monocular/binocular accommodative facility; MEM: monocular estimate method; PRA/NRA: positive/negative relative accommodation; PFV/NFV: positive/negative fusional vergence; NPC: near point of convergence; VF: vergence facility; Δ: prism diopters; D: diopters; cpm: cycles per minute; BO: base-out; BI: base-in).

Dysfunction	Fundamental sign	Complementary sign
Accommodative dysfunctions		
Accommodative insufficiency	Reduced AA: 2.00 D < minimum AA (15–0.25 × age)	MAF < 6 cpm with −2.00 D lenses
		BAF < 3 cpm with −2.00 D lenses
		MEM > 0.75 D
		PRA < 1.25 D
		
Accommodative excess	MAF < 6 cpm with +2.00 D lenses	PRA ≥ 3.50 D
		BAF < 6 cpm with +2.00 D lenses
		MEM < 0.25 D
		NRA < 1.50 D
		
Accommodative infacility	MAF < 6 cpm with ±2.00 D lenses	BAF < 3 cpm with ±2.00 D lenses
		PRA < 1.25 D
		NRA < 1.50 D

Binocular dysfunctions		
Convergence insufficiency	Significant exophoria at near vision (≥6 Δ), greater than far vision	PFV at near ≤ 11/14/3 Δ
		NPC ≥ 6 cm
		VF < 13,4 cpm with 12 Δ base-out prism
		BAF < 3 cpm with +2.00 D lenses
		MEM < 0.25 D
		NRA < 1.50 D
		
Convergence excess	Significant esophoria at near vision (≥1 Δ), greater than far vision	NFV at near ≤ 8/16/7 Δ
		VF < 13.4 cpm with 3 Δ base-in prism
		BAF < 3 cpm with −2.00 D lenses
		MEM > 0.75 D
		PRA < 1.25 D
		
Divergence excess	Significant exophoria at far vision (≥4 Δ), greater than near vision (the difference must be >5 Δ)	PFV at far ≤ 4/10/5 Δ
		PFV at near ≤ 11/14/3 Δ
		NPC ≥ 6 cm
		VF < 13,4 cpm with 12 Δ base-out prism
		BAF < 3 cpm with +2.00 D lenses
		MEM < 0.25 D
		NRA < 1.50 D
		
Basic esophoria	Significant esophoria at far and near vision of equal amount (deviations within 5 Δ of one another are considered equal)	NFV at far ≤ *X*/3/1 Δ and at near ≤ 8/16/7 Δ
		VF < 13.4 cpm with 3 Δ base-in prism
		BAF < 3 cpm with −2.00 D lenses
		MEM > 0.75 D
		PRA < 1.25 D
		
Basic exophoria	Significant exophoria at far and near vision of equal amount (deviations within 5 Δ of one another are considered equal)	PFV at far ≤ 4/10/5 Δ and at near ≤ 11/14/3 Δ
		NPC ≥ 6 cm
		VF < 13,4 cpm with 12 Δ base-out prism
		BAF < 3 cpm with +2.00 D lenses
		MEM < 0.25 D
		NRA < 1.50 D
		
Fusional vergence dysfunction	PFV and NFV reduced at far and near vision or VF < 13.4 cpm with both prisms of the combination used of 12 BO/3 BI	BAF < 3 cpm with ±2.00 D lenses
		PRA < 1.25 D and NRA < 1.50 D

**Table 2:** Symptoms categories encountered in all subjects of the sample.

Symptoms	Number of complaints of patients	
	*n*	%
Visual fatigue	22	20.0
Headache	16	14.5
Dry or gritty eyes	14	12.7
Sore eyes	12	10.9
Blurred vision	11	10,0
Ocular pain	9	8.2
Red eye	7	6.4
Excessive sensitivity to light	6	5.5
Lack of concentration	2	1.8
Excessive blinking	2	1.8
Eye turn noticed	2	1.8
Difficulty in performing schoolwork	1	0.9
Words appearing to move or jump at near vision	1	0.9
Difficulty in focusing from one distance to another	1	0.9
Avoiding near task	1	0.9
Tearing	1	0.9
Pulling eyes	1	0.9
Feeling sleepy	1	0.9
Total	**110**	**100**

**Table 3:** Symptoms related to subjects with refractive dysfunction and without dysfunction.

Dysfunction (*n*)	Number of patients with symptoms	Complaints	
		*N*	%
Without dysfunction (*n* = 55)	**14**	**17**	**15.44**
Visual fatigue		4	3.63
Headache		3	2.72
Ocular pain		3	2.72
Dry or gritty eyes		2	1.82
Blurred vision		1	0.91
Excessive sensitivity to light		1	0.91
Sore eyes		2	1.82
Red eye		1	0.91

Refractive dysfunction (*n* = 59)	**27**	**43**	**39.08**
Headache		9	8.18
Visual fatigue		7	6.36
Blurred vision		6	5.45
Dry or gritty eyes		5	4.55
Excessive sensitivity to light		3	2.72
Sore eyes		3	2.72
Ocular pain		2	1.82
Excessive blinking		2	1.82
Avoiding near task		1	0.91
Tearing		1	0.91
Lack of concentration		1	0.91
Difficulty in focusing from one distance to another		1	0.91
Pulling eyes		1	0.91
Feeling sleepy		1	0.91

**Table 4:** Symptoms related to subjects with accommodative dysfunctions (AE: accommodative excess; AI: accommodative insufficiency).

Dysfunction (*n*)	Number of patients with symptoms	Complaints	
		*N*	%
Accommodative excess (*n* = 13)			
AE suspect (*n* = 1)	**1**	**1**	**0.91**
Red eye		1	0.91
AE high suspect (*n* = 7)	**3**	**5**	**4.55**
Dry or gritty eyes		2	1.82
Ocular pain		1	0.91
Sore eyes		1	0.91
Visual fatigue		1	0.91
AE definite (*n* = 5)	**4**	**4**	**3.63**
Red eye		1	0.91
Sore eyes		1	0.91
Visual fatigue		2	1.82

Accommodative insufficiency (*n* = 3)			
AI suspect (*n* = 3)	**2**	**5**	**4.55**
Blurred vision		1	0.91
Difficulty in performing schoolwork		1	0.91
Dry or gritty eyes		1	0.91
Visual fatigue		2	1.82

Accommodative infacility (*n* = 2)			
Accommodative infacility suspect (*n* = 3)	**1**	**1**	**0.91**
Sore eyes		1	0.91

**Table 5:** Symptoms related to subjects with binocular dysfunctions (CI: convergence insufficiency; CE: convergence excess; DE: divergence excess).

Dysfunction (*n*)	Number of patients with symptoms	Complaints	
		*N*	%
Convergence insufficiency (*n* = 17)			
CI suspect (*n* = 3)	**2**	**3**	**2.72**
Eye turn noticed		1	0.91
Headache		1	0.91
Visual fatigue		1	0.91
CI high suspect (*n* = 4)	**2**	**4**	**3.63**
Dry or gritty eyes		1	0.91
Ocular pain		1	0.91
Red eye		1	0.91
Sore eyes		1	0.91
CI definite (*n* = 10)	**6**	**6**	**5.45**
Excessive sensitivity to light		2	1.82
Dry or gritty eyes		1	0.1
Headache		1	0.91
Ocular pain		1	0.91
Visual fatigue		1	0.91

Convergence excess (*n* = 14)			
CE suspect (*n* = 2)	**0**	**0**	**0**
—			
CE high suspect (*n* = 6)	**2**	**2**	**1.82**
Blurred vision		1	0.91
Headache		1	0.91
CI definite (*n* = 6)	**4**	**5**	**4.55**
Blurred vision		2	1.82
Headache		1	0.91
Sore eyes		1	0.91
Visual fatigue		1	0.91

Divergence excess (*n* = 1)			
DE definite (*n* = 1)	**1**	**1**	**0.91**
Eye turn noticed		1	0.91

Basic exophoria (*n* = 1)			
Basic exophoria definite (*n* = 1)	**1**	**2**	**1.82**
Dry or gritty eyes		1	0.91
Sore eyes		1	0.91

Basic esophoria (*n* = 4)			
Basic esophoria high suspect (*n* = 2)	**1**	**1**	**0.91**
Visual fatigue		1	0.91
Basic esophoria definite (*n* = 2)	**2**	**5**	**4.55**
Lack of concentration		1	0.91
Ocular pain		1	0.91
Red eye		1	0.91
Sore eyes		1	0.91
Visual fatigue		1	0.91

**Table 6:** Symptoms related to subjects with both accommodative and binocular dysfunctions (CI: convergence insufficiency; CE: convergence insufficiency; AI: accommodative insufficiency; AE: accommodative excess; FVD: fusional vergence dysfunction).

Dysfunction (*n*)	Number of patients with symptoms	Complaints	
		*N*	%
CI + AI (*n* = 2)			
CI definite + AI suspect (*n* = 2)	**2**	**2**	**1.82**
Dry or gritty eyes		1	0.91
Words appearing to move or jump at near vision		1	0.91

CI + AE (*n* = 2)			
CI suspect + AE high suspect (*n* = 1)	**1**	**1**	**0.91**
Red eye		1	0.91
CI definite + AE definite (*n* = 1)	**0**	**0**	**0**
—			

CE + AI (*n* = 1)			
CE high suspect + AI suspect (*n* = 1)	**1**	**1**	**0.91**
Red eye		1	0.91

FVD + AI (*n* = 1)			
FVD suspect + AI suspect (*n* = 1)	**1**	**1**	**0.91**
Visual fatigue		1	0.91

**Table 7:** Adjusted estimation of the association between visual symptomatology and all diagnosed dysfunctions including the different grades of dysfunctions according to the number of clinical signs used (RD: refractive dysfunction; AD: accommodative dysfunction; BD: binocular dysfunction; CI: confidence interval).

	OR					
	Raw	CI 95%	*p*	Adjusted^†^	CI 95%	*p*
RD	2.47	1.12–5.47	0.026^*∗*^	NA	NA	NA

AD suspect, high suspect, and definite	4.60	1.49–14.18	0.008^*∗*^	2.34	0.55–9.97	0.249
AD high suspect and definite	3.91	1.15–13.23	0.029^*∗*^	2.20	0.44–11.05	0.340
AD definite	11.71	1.21–113.81	0.034^*∗*^	—	—	0.999

BD suspect, high suspect, and definite	3.84	1.58–9.35	0.003^*∗*^	3.35	1.03–10.91	0.045^*∗*^
BD high suspect and definite	4.28	1.69–10.85	0.002^*∗*^	4.10	1.12–15.02	0.033^*∗*^
BD definite	6.83	2.20–21.21	0.001^*∗*^	8.79	1.59–48.65	0.013^*∗*^

AD + BD	14.64	1.57–136.33	0.018^*∗*^	8.79	0.84–91.49	0.069

^†^OR adjusted by an uncorrected refractive error.

^*∗*^Significant association.
